# A review of the pathogenic mechanism and clinical management progress of in-stent calcification restenosis

**DOI:** 10.3389/fcvm.2026.1788890

**Published:** 2026-05-29

**Authors:** Xiangchen Xia, Fei Luo, Jianchun Li, Quanyi Liu, Jianzhong Pang, Fuyun Jia, Qiang Xu, Chao Peng

**Affiliations:** 1 Department of Cardiovascular, Xingyi People’s Hospital, Guizhou, China; 2 Department of Cardiology, The Second Affiliated Hospital of Tianjin University of Traditional Chinese Medicine, Tianjin, China

**Keywords:** calcification, calcification mechanism, extracorporeal shock wave lithotripsy, in-stent restenosis, interventional therapy, neoatherosclerosis

## Abstract

Calcific in-stent restenosis (ISR) is a challenging complication of coronary percutaneous coronary intervention (PCI) that severely impairs long-term prognosis. Calcified lesions restrict stent expansion and exacerbate neointimal hyperplasia and neoatherosclerosis, thereby increasing the risk of ISR. Recent advances in interventional and imaging technologies have deepened insights into its molecular mechanisms, biomechanical disorders, inflammatory responses, and calcification morphology-related risks. High-resolution intravascular imaging (OCT/IVUS) enables precise evaluation of calcification characteristics, while novel therapies including intravascular lithotripsy (IVL), orbital atherectomy, and hybrid ablation strategies have revolutionized the management of coronary calcified ISR. This review systematically summarizes the pathophysiological mechanisms, imaging evaluation, and advances in the clinical management of coronary in-stent calcific restenosis, proposes a clinical treatment algorithm, and outlines future research directions, aiming to provide evidence-based guidance for clinical practice.

## Introduction

In-stent calcific restenosis (ISR) represents one of the most formidable challenges in coronary interventional therapy, characterized by a complex etiopathogenesis and difficult clinical management. Calcification, a hallmark pathological feature of atherosclerosis, critically impairs adequate stent expansion and predisposes to restenosis (1). Advancements in intracoronary imaging, particularly intravascular ultrasound (IVUS) and optical coherence tomography (OCT), have provided profound insights into the morphological characteristics (e.g., macro-calcification, punctate calcification) and spatial distribution of calcified plaques, clarifying their impact on restenosis risk (2–4). The aberrant mechanical stress induced by calcification is a pivotal driver of stent dysfunction, altering vessel wall biomechanics and compromising stent-vessel interaction, which leads to complications such as underexpansion and malapposition (5, 6).

Calcification contributes to the restenotic process through multiple interrelated pathways. Primarily, calcified plaques may fracture during stent deployment, creating sharp edges that perpetuate endothelial injury and stimulate neointimal hyperplasia (7–9). Secondly, calcified regions impede both the elution and transmural penetration of antiproliferative drugs from drug-eluting stents (DES), resulting in subtherapeutic local drug concentrations and inadequate suppression of neointimal growth (10, 11). Furthermore, calcification potentiates inflammation and exacerbates oxidative stress, thereby fostering the development of neoatherosclerosis (12–14). Clinically, the density and pattern of calcification correlate significantly with restenosis risk. High-density calcification (≥800 Hounsfield units) may confer relative stability and lower restenosis rates, whereas lower-density calcification is associated with a higher incidence of ISR (15).

Conventional treatment modalities, including high-pressure non-compliant balloon angioplasty, rotational atherectomy, and drug-coated balloons (DCB), are often limited by inadequate lesion preparation in severe calcification, suboptimal stent expansion, and the risk of vascular trauma or distal embolization (16, 17). Even with high-pressure balloons, sufficient expansion remains challenging in heavily calcified lesions, while DCB efficacy is compromised by impaired drug transfer in such settings (18). Novel technologies have emerged to address these limitations. Intravascular lithotripsy (IVL) selectively disrupts calcific plaque with minimal adjacent soft-tissue injury, enhancing lumen gain and procedural safety (19–21). Complementary techniques (e.g., laser balloon angioplasty, orbital atherectomy, excimer laser coronary angioplasty) and hybrid combination therapies allow for optimized management of complex lesions. Next-generation devices (e.g., Lithix, Javelin systems) hold promise for more precise and personalized treatment for refractory calcific ISR (22–24). This review systematically synthesizes the pathophysiological mechanisms, imaging evaluation, and clinical management advances of calcific ISR, aiming to guide personalized clinical practice.

## Pathophysiological mechanisms of in-stent calcification restenosis

### Molecular biological mechanisms of calcification

Vascular calcification involves the regulation of multiple cellular processes and the regulation of multiple signaling pathways. Under physiological conditions, vascular smooth muscle cells (VSMCs) maintain a contractile phenotype; upon exposure to injury or stress, VSMCs transition to a synthetic phenotype and even undergo osteoblast-like differentiation, which is regulated by BMP and Wnt/β-catenin pathways (25–27). FGFR3 gene mutations are involved in vascular calcification by affecting VSMC biological behaviors (28).

Calcification is accompanied by cellular apoptosis and inflammatory activation. The NF-*κ*B signaling pathway mediates inflammatory factor release and extracellular matrix remodeling,while cysteine proteases (CTSs) participate in vascular calcification via proteolysis and signal transduction (29), Lipoprotein(a) [Lp(a)] promotes vascular calcification through pro-inflammatory and pro-atherosclerotic effects, and its targeted antisense oligonucleotide therapy shows promise (30).

**Figure 1:**
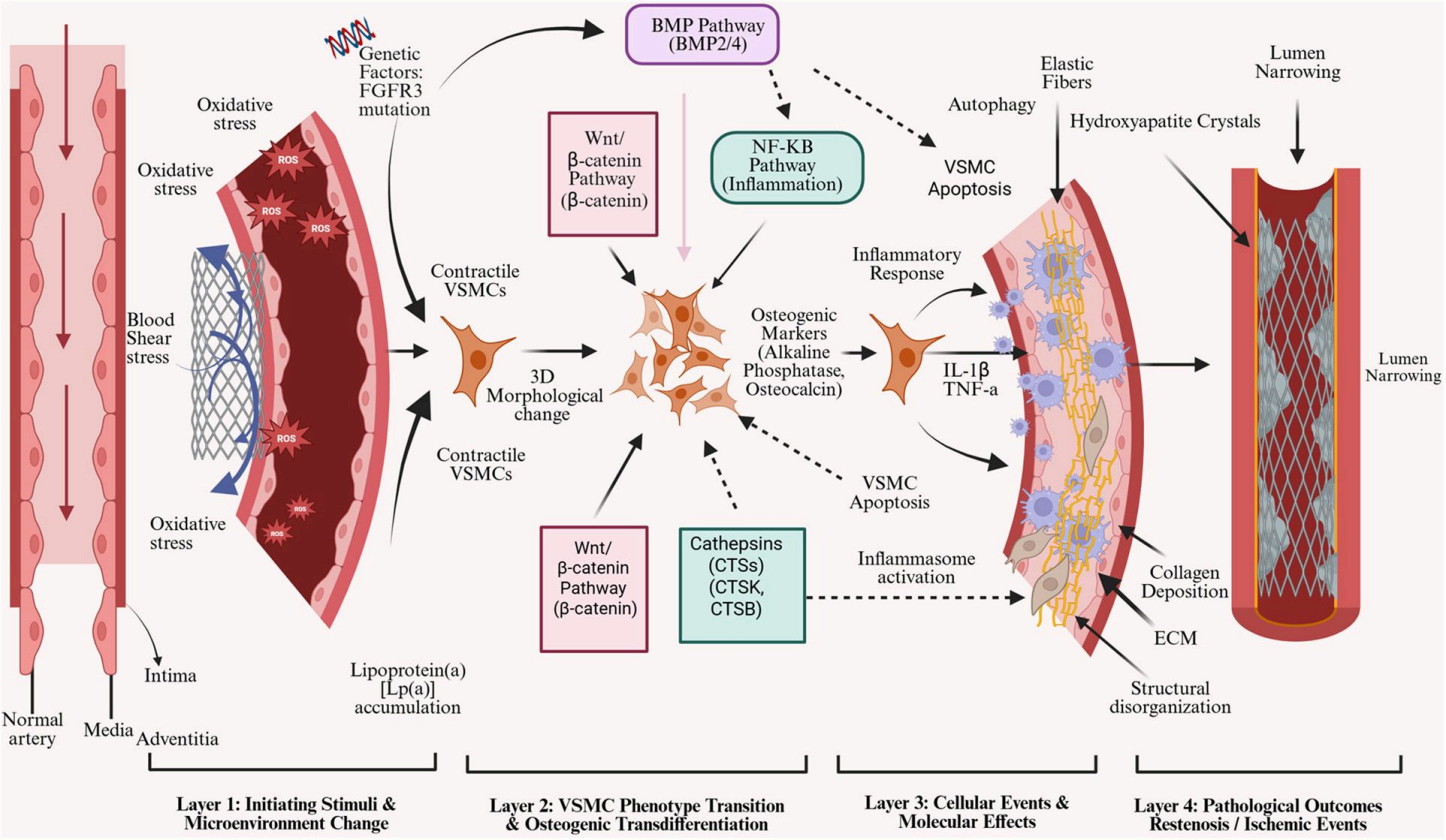
Schematic diagram of the multi-stage molecular and cellular mechanisms underlying the progression of vascular lesions. Illustration of the continuous process of vascular disease from initial stimulus to pathological outcome: (1). Initial stimulus and microenvironment alteration stage: Normal arteries are affected by shear stress, oxidative stress, lipoprotein **(a)** accumulation, and FGFR3 mutations, leading to disruption of the vascular wall microenvironment. (2). VSMC phenotype transition and osteogenic transdifferentiation stage: Regulated by BMP, the Wnt/*β*-catenin pathway, and cathepsins (CTSs), contractile vascular smooth muscle cells (VSMCs) undergo phenotype transition and osteogenic transdifferentiation, expressing alkaline phosphatase and other osteogenic markers; (3). Cellular events and molecular effects stage: The NF-*κ*B pathway mediates the activation of inflammasomes, the release of inflammatory factors (IL-1β, TNF-α), and VSMC apoptosis, accompanied by disruption of the extracellular matrix (ECM) structure; (4). Pathological outcome stage: These processes ultimately lead to hydroxyapatite deposition and lumen narrowing, culminating in restenosis or ischemic events.

In summary, the molecular biological mechanisms of in-stent calcification involve the abnormal activation of various signaling pathways, regulation by genetic factors, and inflammatory responses, with the interaction of these factors collectively promoting the formation and progression of calcified lesions (Figure 1).

### Types of calcification and their impact on stent function

Vascular calcification is a critical pathological substrate for in-stent restenosis, with its morphological characteristics directly influencing stent mechanical performance and biological response (31). Based on distribution, calcification is categorized into macro-calcification, punctate calcification, and calcified nodules, each impairing stent function through distinct mechanisms (7, 32, 33). Macro-calcification, typically presenting as continuous calcific plaques, exhibits high rigidity that limits stent expansion, preventing full stent apposition. This incomplete expansion increases local mechanical stress and promotes stent strut metal fatigue, ultimately raising the risks of stent fracture and restenosis (34). Studies indicate that stress concentration on stent struts in macro-calcified areas is 3–5 times higher than in non-calcified areas after cobalt-chromium alloy stent implantation, serving as a significant predictor for late stent fracture (35). Furthermore, the resultant altered local hemodynamics increases the risk of platelet activation and thrombosis (32).

**Figure 2:**
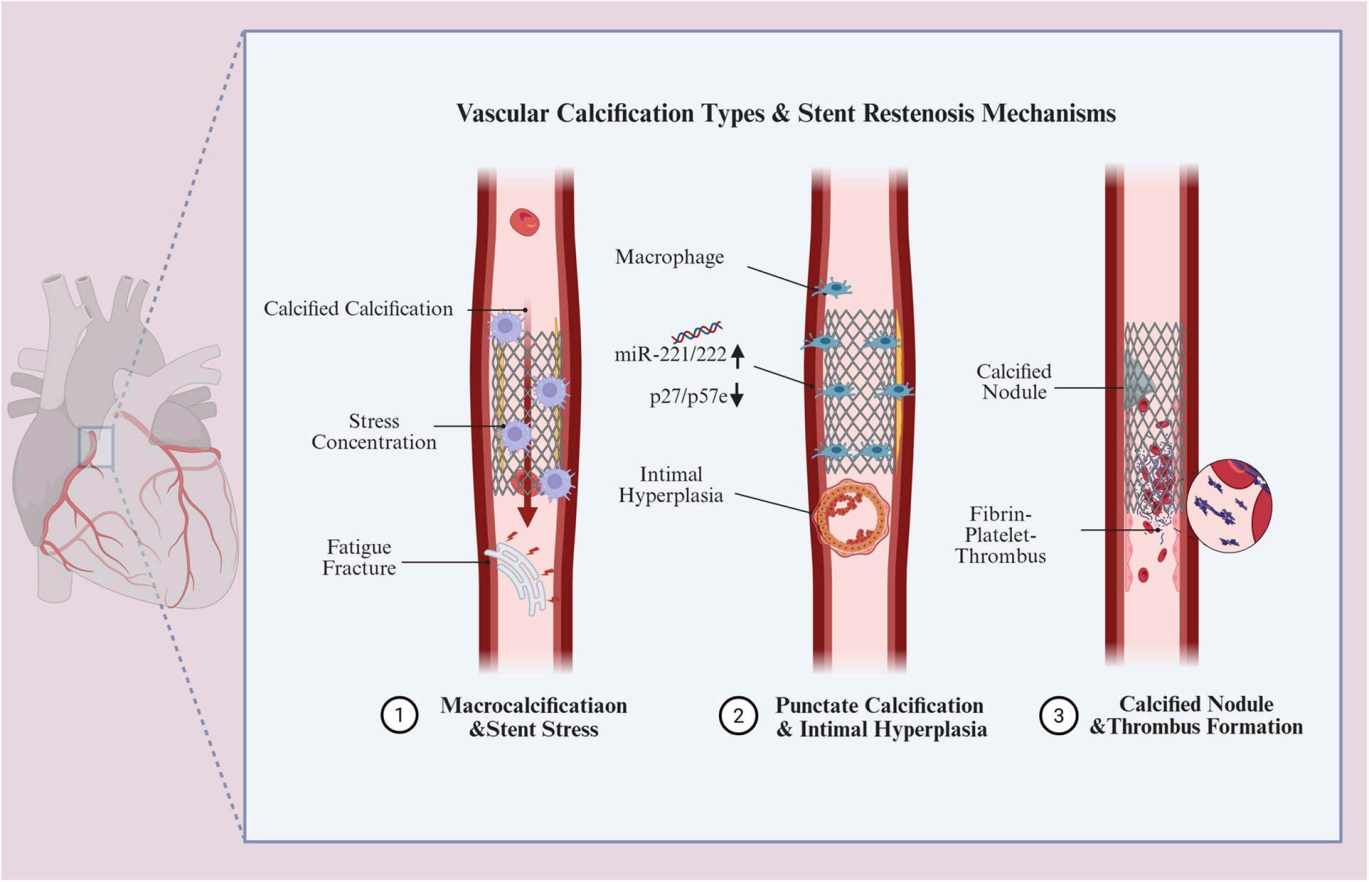
Schematic diagram of the mechanisms of vascular calcification types and in-stent restenosis. Illustration of three mechanisms of stent restenosis associated with vascular calcification: (1). Left image (large calcification and stent stress): Extensive calcified areas in the vessel wall result in localized stress concentration on the stent, thereby inducing stent fatigue fracture; (2). Middle image (punctate calcification and intimal hyperplasia): Punctate calcification, coupled with macrophage infiltration, the upregulation of miR-221/222, and the downregulation of p27/p57, drives vascular intimal hyperplasia; (3). Right image (calcified nodules and thrombosis): Calcified nodules induce localized hemodynamic disturbances, ultimately promoting fibrin-platelet thrombosis.

In contrast, punctate calcifications, though small (<3 mm), carry substantial pathological significance. OCT studies reveal they are often accompanied by active inflammation, characterized by increased macrophage infiltration and elevated levels of pro-inflammatory factors (e.g., IL-6, TNF-α) (36). This microenvironment stimulates vascular smooth muscle cell (VSMC) phenotypic switching to a synthetic state, promoting excessive intimal hyperplasia. Molecular studies show elevated miR-221/222 expression in these regions accelerates VSMC proliferation by inhibiting cell cycle inhibitors p27Kip1 and p57Kip2 (37). The most destructive type, calcified nodules, is characterized by sharp, protruding structures (38). They directly compress the lumen, causing a “tenting effect” that separates stent struts from the vessel wall. Hemodynamic simulations show turbulence and low shear stress zones downstream of nodules promote platelet aggregation and fibrin deposition (39), while pathological studies confirm their surfaces are often covered with fibrin-platelet thrombus (30). Clinically, managing nodular lesions is challenging; traditional high-pressure balloon dilation carries a 4.7% perforation risk, whereas intravascular lithotripsy (IVL) increases the complete stent expansion rate to 92% and is associated with significantly fewer complications (40, 41). A meta-analysis of 22,739 lesions confirms these risks: macro-calcification significantly increases 3-year target vessel failure risk (HR: 1.37, 95% CI: 1.19–1.58), while calcified nodules carry an even higher re-intervention risk (OR: 3.58, 95% CI: 1.33–9.65) (42). Coronary calcification density ≥800 Hounsfield Units (HU) is also associated with an altered risk of in-stent restenosis, closely related to plaque stability (43) (Figure 2).

### Interaction between biomechanical factors and calcification

The interaction between vascular calcification and biomechanical factors is an important mechanism in the pathogenesis and progression of in-stent restenosis. The formation of calcified plaques significantly increases the rigidity of the vascular wall, leading to a decrease in vascular compliance, thereby altering the local hemodynamic environment. Studies have shown that calcific deposition can significantly impact the progression of cardiovascular diseases and the effectiveness of interventional treatments (44). The presence of calcified plaques causes localized high shear stress and stress concentration in the vascular wall induced by hemodynamic forces. This abnormal mechanical environment further promotes the deposition of calcium salts and the progression of plaques, forming a vicious cycle (45). Moreover, the uneven distribution of calcified plaques can lead to regional differences in the mechanical properties of the vascular wall. This mechanical heterogeneity leads to uneven local stress distribution and localized stress concentrations, which promote plaque instability and rupture, serving as a critical predisposing factor (46).

After stent implantation, the interaction between the calcified plaque and the stent generates complex biomechanical effects. The mechanical stiffness and brittleness of calcified plaques hinder full stent expansion in the calcified region, leading to local deformation or even fracture of the stent (47). Experimental studies have demonstrated significant heterogeneity in the mechanical stiffness of calcified nodules. Some nodules exhibit notable compressive resistance even at low strain levels, a characteristic that directly affects the stent's wall apposition (47). Furthermore, the mismatch between the stent and the calcified plaque can lead to localized stress concentration, thereby damaging the vascular endothelium, triggering inflammatory responses, and promoting neointimal hyperplasia (48). Additionally, the geometry of the calcified plaque also impacts the effectiveness of stent expansion. Circular or arcuate calcified deposits exert stronger resistance to balloon expansion, predisposing the stent to incomplete expansion (49).

**Figure 3:**
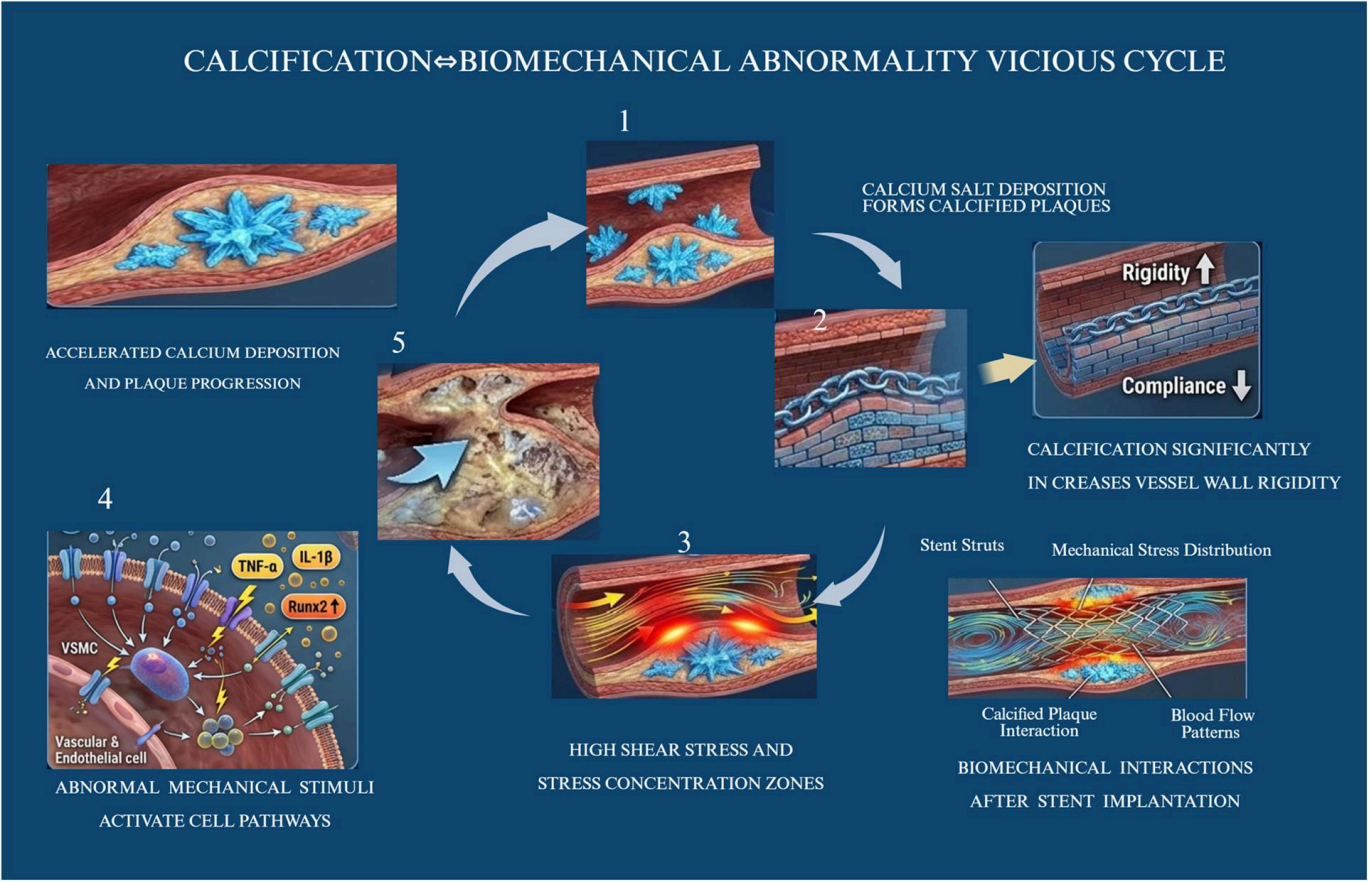
The vicious cycle mechanism of vascular calcification and biomechanical abnormalities. The positive feedback mechanism of vascular calcification and biomechanical disorder: (1). Calcium salt deposition forms calcified plaques; (2). Calcification significantly increases vascular wall stiffness and decreases compliance; (3). After stent implantation, the biomechanical interaction between calcified plaques and the stent generates regions of high shear stress and stress concentration; (4). Abnormal mechanical stimulation activates vascular cell pathways (e.g., the release of TNF-α and IL-1β, and the upregulation of Runx2); (5). These pathways further accelerate calcium deposition and plaque progression, forming a self-perpetuating cycle.

**Figure 4:**
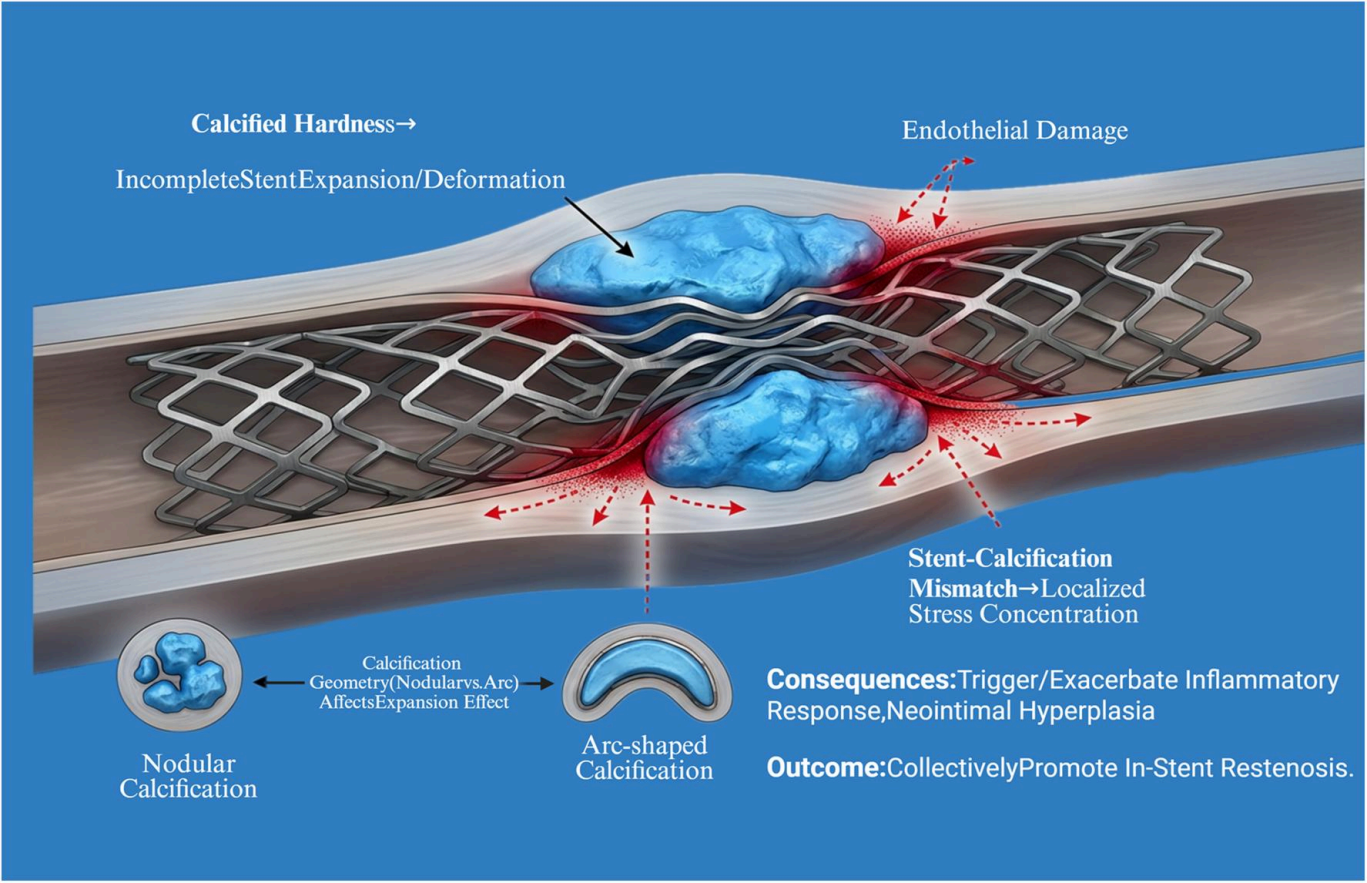
The mechanism of vascular calcification on stent implantation and restenosis. Mechanism by which vascular calcification drives in-stent restenosis: The rigidity of calcified plaques, characterized by nodular or arc-shaped morphologies that impede stent expansion, results in incomplete or asymmetric stent deployment, while the mismatch between the stent and the calcified lesion induces local stress concentration and endothelial injury, thereby triggering or exacerbating the inflammatory response and neointimal hyperplasia, which ultimately promotes the development of in-stent restenosis.

Biomechanical imbalance plays a critical role in calcification-related vascular injury. Abnormal biomechanical stimuli can activate mechanosensitive signaling pathways in vascular endothelial cells and smooth muscle cells, thereby promoting the release of inflammatory factors and inducing osteogenic differentiation (50). Studies indicate that the high-stress environment in calcified areas can upregulate the expression of osteogenic markers such as Runx2, accelerating the calcification process (48). Concurrently, mechanical stress can regulate the polarization of macrophages, modulating the local inflammatory microenvironment (51).This coupling of mechanical stimuli and biological responses forms a positive feedback loop: calcification leads to biomechanical abnormalities, which further promote pathological progression, ultimately resulting in in-stent restenosis (32). Elucidating this complex mechanism is essential for the development of targeted therapeutic strategies (Figures 3, 4).

### Inflammatory response and immune mechanisms

The occurrence of in-stent calcification restenosis is closely related to chronic inflammatory responses. Studies have shown that calcified plaque regions are often accompanied by chronic inflammatory cell infiltration, with macrophages and T lymphocytes playing key roles in the formation and stabilization of calcified plaques (39). These immune cells promote the phenotypic transformation and migration of vascular smooth muscle cells (VSMCs) by secreting pro-inflammatory cytokines (such as IL-6, TNF-α) and chemokines, leading to intimal hyperplasia and collagen deposition. Notably, the miR-221/222 cluster plays an important role in regulating VSMC proliferation and inflammatory responses. The elevated expression levels of the miR-221/222 cluster are significantly associated with the severity of in-stent restenosis (37).

Inflammatory mediators also influence the calcification process through various pathways. On the one hand, macrophages promote extracellular matrix remodeling by releasing matrix metalloproteinases (MMPs), creating a favorable environment for calcium salt deposition; on the other hand, inflammatory factors can upregulate the expression of osteogenic transcription factors (such as Runx2 and BMP-2), inducing vascular smooth muscle cells (VSMCs) to differentiate into osteoblast-like cells (37, 52). Clinical imaging studies further confirm that an increase in the fat attenuation index (FAI) of the adipose tissue surrounding the lesion before stent implantation (reflecting enhanced local inflammatory activity) is significantly associated with the risk of subsequent restenosis (52).

**Figure 5:**
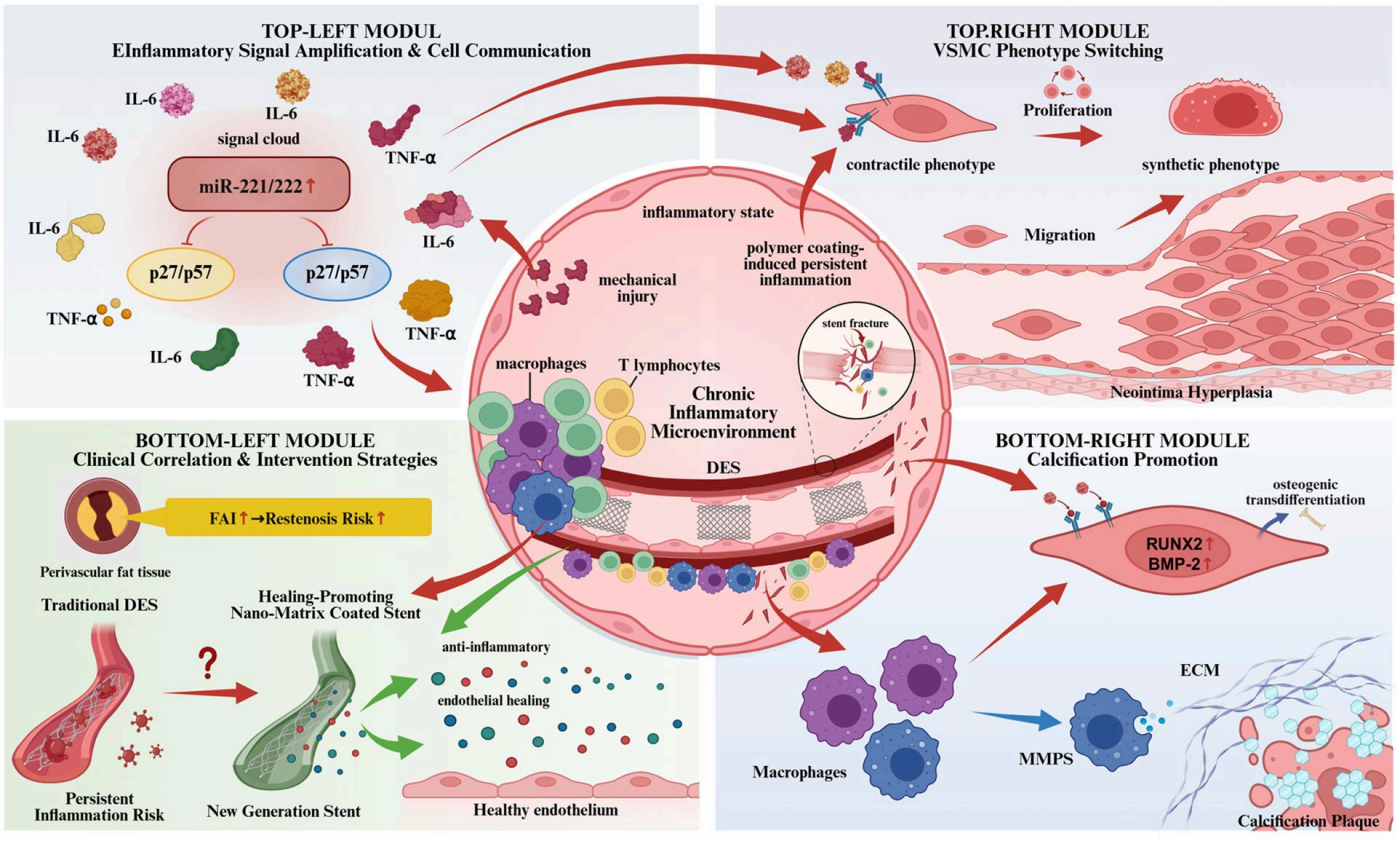
Multi-module regulatory mechanisms of the chronic inflammatory microenvironment associated with drug-eluting stents (DES). Mechanical injury, polymer coating stimulation, and stent fracture after DES implantation induce infiltration of immune cells such as macrophages and T lymphocytes, forming a persistent chronic inflammatory microenvironment that provides the initial drive for the pathological progression of in-stent restenosis, demonstrating the regulatory effects of four modules: (1). Upper left module: Amplification of inflammatory signals and immune communication (signaling drivers of restenosis): Inflammatory factors such as IL-6 and TNF-α form a “signal cloud”, amplifying inflammatory signals through the upregulation of miR-221/222 and the inhibition of pathways such as those involving p27/p57, strengthening communication between immune cells and local cells, providing continuous inflammatory-immune stimulation for subsequent pathological processes. (2). Upper right module: Inflammation-mediated VSMC phenotypic switching (core pathological link of restenosis): Inflammatory-immune signals drive vascular smooth muscle cells (VSMC) to switch from a contractile phenotype to a synthetic phenotype, accompanied by enhanced proliferation and migration, ultimately leading to neointimal hyperplasia—one of the hallmark pathological changes of in-stent restenosis. (3). Lower right module: Inflammation-induced vascular calcification (pathological synergistic link of restenosis): The chronic inflammatory microenvironment activates the osteogenic transdifferentiation of VSMC (upregulation of Runx2 and BMP-2), while macrophages release matrix metalloproteinases (MMPs) that disrupt the structure of the extracellular matrix (ECM), promoting the formation of calcified plaques and further exacerbating vascular stenosis. (4). Lower left module: Inflammation-related clinical associations and interventions (risk regulation of restenosis): Elevated fat attenuation index (FAI) of perivascular adipose tissue is positively correlated with the risk of inflammation-driven restenosis; traditional DES increases the risk of restenosis due to persistent inflammatory stimulation, while the new generation of healing-promoting nanomatrix-coated stents targets the inflammatory-immune mechanisms through anti-inflammatory and endothelial repair strategies to reduce the incidence of restenosis.

The stenting procedure itself can exacerbate the inflammatory response. The mechanical damage to the vessel wall caused by stent expansion can activate damage-associated molecular patterns (DAMPs), which in turn trigger an innate immune response (39). Although drug-eluting stents (DES) can inhibit neointimal hyperplasia, the persistent inflammatory state observed in some patients may actually promote late-stage calcification and restenosis (39). A novel healing-promoting nanoscale matrix-coated drug-eluting stent shows advantages in simultaneously suppressing inflammation and promoting endothelialization by regulating the interactions among various vascular cells, such as endothelial cells, smooth muscle cells, and immune cells. This provides new insights for improving the long-term outcomes of stent therapy (53). In addition, mechanical complications such as stent fracture can continuously stimulate local inflammatory responses, thereby increasing the risk of aneurysm formation and restenosis (54). These findings emphasize the importance of considering inflammation regulation comprehensively in stent design and clinical application (Figure 5).

### Factors related to interventional procedures

Insufficient stent expansion is a pivotal procedural factor leading to adverse outcomes, including in-stent restenosis (ISR), in percutaneous coronary intervention (PCI) (55, 56). This critical limitation arises when a stent fails to achieve the diameter of the adjacent reference vessel segment, resulting in inadequate lumen gain. Key procedural determinants of such under-expansion include severe coronary artery calcification (CAC), inadequate lesion preparation, and suboptimal stent deployment techniques (55). Calcified plaques, particularly behind previously implanted stents, present a significant challenge as they resist adequate expansion even with high-pressure balloon dilation, often necessitating advanced, higher-risk techniques (55). Furthermore, procedural complications such as stent malposition, multilayer stent overlap, or stent fracture—influenced by stent design, vascular anatomy (e.g., angulation), and deployment technique—are also associated with an increased risk of restenosis and major adverse cardiovascular events (57).

Managing severely calcified coronary lesions using traditional techniques is fraught with limitations that may contribute to ISR. Although high-pressure balloon dilation (HPBD) and rotational atherectomy (RA) aim to modify calcified plaques to facilitate stent expansion, both procedures carry inherent risks. Severe CAC increases procedural complexity and elevates the risk of complications, including vascular injury, stent rupture, and coronary perforation (58, 59). RA, although effective for lesion preparation, is associated with high procedural complexity, prolonged operation time, and risks of vascular injury (60). HPBD, especially for calcified in-stent restenosis, has a higher failure rate; case reports indicate that severe calcification can still lead to inadequate stent expansion even after intervention, contributing to very late ISR (61). The choice and execution of these conventional techniques significantly impact outcomes, underscoring that suboptimal lesion preparation remains a key procedural factor in the development of calcific ISR.

### Impact of calcification on DES efficacy

Calcified plaques critically impair the efficacy of drug-eluting stents (DES) in coronary interventions by creating substantial barriers to optimal drug pharmacokinetics. Primarily, the dense inorganic matrix of calcification acts as a physical barrier, hindering the uniform release and transmural penetration of antiproliferative drugs (e.g., sirolimus, paclitaxel) from the stent coating, leading to insufficient local drug concentrations and suboptimal inhibition of neointimal hyperplasia (62). This barrier effect is exacerbated by the heterogeneous structure of calcified nodules, which are prevalent in severe lesions and associated with lower drug bioavailability (63). Furthermore, the calcified microenvironment itself alters drug dynamics; negatively charged hydroxyapatite crystals can adsorb positively charged drugs, resulting in abnormal local accumulation rather than effective diffusion into the vessel wall (64, 65). Alterations in pH and enzymatic activity within calcified plaques may also accelerate drug degradation (66). The resultant inefficient drug transport creates localized “drug desert” areas, particularly at stent edges, which become high-risk sites for restenosis (67).

The impact of calcification extends beyond passive barriers to active biological and mechanical processes that counteract DES function (68). The high rigidity of calcified plaques frequently results in stent under-expansion and malapposition. The micro-gaps formed between inadequately apposed stent struts and the vessel wall prevent direct drug contact with the target tissue, a phenomenon observed via OCT in up to 42.2% of calcified lesions and associated with a 3.39-fold increased restenosis risk (10, 69). Concurrently, the local inflammatory response activated by calcification—characterized by upregulated IL-6 and TNF-α—promotes vascular smooth muscle cell proliferation via pathways such as the NF-κB pathway, which can partially offset the effect of antiproliferative drugs (13, 70). This synergistic effect of mechanical obstruction and enhanced inflammatory-proliferative signaling significantly diminishes the long-term efficacy of DES in calcified coronary lesions (71).

## Imaging assessment of in-stent calcification restenosis

### Optical coherence tomography (OCT)

Optical coherence tomography (OCT) is a high-resolution intravascular imaging technology that demonstrates significant advantages in the assessment of in-stent restenosis (ISR) due to calcification. Its axial resolution ranges from 10 to 15 μm, allowing for precise identification of the morphology, thickness, and spatial distribution of calcified plaques. Research shows that OCT can clearly differentiate between macrocalcification (calcification arc >180°) and punctate calcification (calcification arc <90°). Research shows that OCT can clearly differentiate between macrocalcification (calcification arc >180°) and punctate calcification (calcification arc <90°) (72); however, lesions with a calcification arc between 90° and 180° lack a standardized classification. This classification is crucial for clinical risk assessment and the development of treatment strategies. For example, macrocalcification is often associated with more severe adverse outcomes of stent expansion, while punctate calcification may respond better to interventional treatment. Additionally, OCT can quantify calcification burden, which refers to the composite measure of parameters such as the length, arc, and thickness of calcified plaques; the calcification score calculated from these parameters can be used to predict whether stent expansion is adequate (73).

OCT has unique value in the differential diagnosis of calcified plaque subtypes. For example, research has classified calcified plaques into three subtypes using OCT: eruptive calcified nodules, superficial calcified plates, and calcified protrusions. The study found that eruptive calcified nodules are more likely to lead to edge dissection of the stent (47.4% vs. 17.5%) and poor stent apposition (94.7% vs. 58.7%), while superficial calcified plates are associated with a smaller minimum stent area (4.72 ± 1.37 mm^2^) (74). These findings suggest that OCT-guided analysis of calcified subtypes can optimize the selection of interventional devices; for instance, when targeting eruptive calcified nodules, cutting balloons or intravascular lithotripsy (IVL) should be prioritized (75).

Clinical evidence further supports the role of OCT in predicting ISR risk. A study on intracranial arterial stenosis found that macro-calcification detected by OCT was significantly associated with ISR risk (42.86% vs. 11.11%, *p* = 0.0375), Furthermore, the ISR incidence was higher in patients with macrocalcification compared to those with punctate calcification (77.78% vs. 22.22%, *p* = 0.03). In addition, OCT can also evaluate the effectiveness of calcification modification techniques (such as IVL). After calcification rupture, the lumen area of the stent significantly increased (8.0 mm² vs. 7.1 mm^2^, *p* = 0.004). Ruptures are more likely to occur in lesions with larger calcification arcs, superficial calcification, and non-nodular calcification (72). These data indicate that OCT can not only guide preoperative calcification modification strategies but also optimize stent implantation outcomes through real-time intraoperative monitoring, thereby reducing ISR risk.

Overall, with its high resolution and quantitative analysis capabilities, OCT has become an indispensable tool in the interventional treatment of calcified lesions. In the future, with the integration of artificial intelligence algorithms, the application of OCT in the assessment of calcified lesions and personalized treatment will be further enhanced (2).

### Intravascular ultrasound (IVUS)

Intravascular ultrasound (IVUS) serves as a key intraluminal imaging tool, playing a central role in the assessment and management of in-stent restenosis (ISR). IVUS can provide high-resolution images of the cross-section of the vessel wall, allowing for precise evaluation of the volume, distribution, and extent of calcified plaques, which is crucial for guiding complex interventional procedures (76). For example, IVUS can clearly identify superficial circumferential calcification that leads to incomplete stent expansion, providing direct evidence for subsequent plaque modification strategies such as atherectomy (77). Furthermore, IVUS is highly valuable for diagnosing stent fractures, a rare but serious complication, as it can confirm the diagnosis, identify possible mechanisms, and evaluate the outcomes of subsequent balloon angioplasty (78).

IVUS has irreplaceable advantages in measuring and optimizing stent expansion, and is key to predicting the risk of restenosis. Inadequate stent expansion is a significant and challenging issue in percutaneous coronary intervention (PCI), closely associated with increased risks of ISR, in-stent thrombosis, and myocardial infarction (56). IVUS can precisely measure parameters such as the minimum lumen area and stent symmetry after stent implantation, allowing for a quantitative assessment of the adequacy of stent expansion. Guided by these IVUS measurements, operators can more accurately select balloon sizes and expansion pressures to optimize stent implantation outcomes. A large international clinical trial (IMPROVE trial) focused on complex coronary lesions (including severe calcification, ISR, etc.) aims to evaluate the clinical impact of IVUS guidance compared to angiography-only guidance on improving target vessel failure rates. This trial highlights the importance of IVUS in complex and high-risk lesions (79).

Combining optical coherence tomography (OCT) can further enhance the diagnostic accuracy for calcified lesions, particularly calcified nodules. Calcified nodules are a unique type of calcification associated with higher rates of target lesion revascularization, and this increase in revascularization rates is particularly related to inadequate stent expansion (76). OCT, with its higher resolution, can more accurately assess calcification thickness, surface irregularity (such as eruptive vs. non-eruptive calcified nodules), and minor stent malapposition (76), while IVUS has advantages in assessing the area of the external elastic membrane, deep calcification, and overall plaque burden. Additionally, the combined use of both can achieve complementary advantages. For example, in the treatment of left main calcified lesions, the combined use of OCT and IVUS to confirm the presence of significant calcification, assess lesion severity, and guide the selection of appropriate calcification modification techniques (such as cutting balloons, rotational atherectomy, or intravascular lithotripsy) has become a standard procedure (59). This multimodal imaging strategy aids in developing more individualized treatment plans, potentially improving patients' long-term prognosis.

### Computed tomography angiography (CTA)

As a non-invasive imaging technique, Computed Tomography Angiography (CTA) holds significant value in assessing in-stent calcific restenosis. Through high-resolution three-dimensional reconstruction, CTA can accurately quantify the density of calcified plaques (expressed in Hounsfield Units, HU) and their spatial distribution, providing critical information for preoperative planning (80). Combining hemodynamic analysis can further enhance the predictive efficacy of CTA. By eliminating stent metal artifacts, Virtual Intravascular Endoscopy (VIE) technology improves lumen visibility to 91.76%—an increase of 41.2 percentage points from the baseline of 50.59%—and achieves a diagnostic accuracy of 97.43%, surpassing the 95.3% observed with traditional CTA (81). Additionally, new technologies such as Photon Counting Detector CT (PCD-CT) improve the accuracy of stent lumen diameter measurements by reducing “blooming” artifacts. UHR-Bv72 images with a resolution of 0.2 mm show a stent diameter of 2.17 mm, while traditional CT shows 2.1 mm, with a statistically significant difference (*p* < 0.001). This technology improves the diagnostic accuracy for small stents (<3 mm) to 88% (82). Recent studies have also found that the Fat Attenuation Index (FAI) surrounding the stent can serve as an inflammatory marker to predict restenosis. At a Fat Attenuation Index threshold of −69.6 HU, the Area Under the Curve (AUC) for predicting restenosis increases by 0.064 (*p* = 0.001) (15, 83), offering a novel dimension for risk assessment. These technological advancements make CTA an increasingly reliable alternative to invasive angiography, especially demonstrating important clinical value in follow-up monitoring (84).

### Other imaging technologies

In addition to intravascular ultrasound and optical coherence tomography, other imaging technologies also demonstrate unique value in evaluating in-stent calcification restenosis. Magnetic resonance imaging (MRI) provides a new perspective for exploring the biological characteristics of calcification, where calcified plaques typically appear as low-signal areas on MRI, aiding in the identification of their presence and extent. Molecular imaging techniques, through specific probes targeting calcification or inflammatory processes, can reveal the biological activity of calcified plaques.For instance, molecular imaging can assess the inflammatory status of plaques and microcalcification development. These characteristics are closely related to plaque instability and the risk of restenosis (85). These technologies extend from mere morphological assessment to functional and biological evaluations, deepening the understanding of the pathophysiological mechanisms of calcification restenosis (86).

Dynamic imaging techniques are crucial for assessing the evolution of calcified lesions and treatment responses. Three-dimensional imaging technologies, such as cone beam computed tomography (CBCT), can provide valuable information about plaque characteristics, particularly calcification features that may interfere with the effectiveness of angioplasty (87). For example, CBCT can identify calcification barriers or dense calcified areas that impede complete stent expansion. The presence of such barriers is significantly associated with residual stenosis after stent implantation. By evaluating maximum plaque thickness, calcification barriers, and other characteristics through preoperative CBCT, it is possible to predict whether there will be circular or elliptical expansion post-stent implantation and whether significant residual stenosis exists, thus optimizing surgical planning (87).

## Treatment strategies and advances for in-stent calcification restenosis

### Traditional treatment methods

The traditional treatment methods for in-stent restenosis (ISR) primarily include mechanical approaches such as high-pressure non-compliant balloon dilation and rotational atherectomy. High-pressure non-compliant balloons disrupt calcified plaques through mechanical expansion force, but their effectiveness is limited in cases of severe calcification, and they may lead to complications such as vascular injury or stent fracture (61). Studies have shown that relying solely on balloon dilation for severe calcified lesions often fails to achieve ideal lumen gain, with postoperative restenosis rates still as high as 10%–20% (55). Rotational atherectomy removes calcified plaques using a high-speed rotating burr; however, this technique is complex, requires longer operation times, and carries risks such as vascular perforation and the no-reflow phenomenon (88). A meta-analysis involving 2,120 patients indicated that the average operation time for rotational atherectomy was 27.9 min longer than that for intravascular lithotripsy (IVL), with greater amounts of contrast agent used (55). Furthermore, rotational atherectomy is particularly challenging in managing calcified lesions in multilayer stents or overlapping stent areas, which may lead to severe complications such as stent fractures or wire entrapment (89).

Drug-coated balloons (DCB) represent another treatment option for ISR, which inhibit neointimal hyperplasia by locally releasing anti-proliferative drugs and have been widely applied. However, multiple studies have shown that the efficacy of DCB in calcified lesions is suboptimal (90). In the coronary artery field, the target lesion revascularization rate (TLR, or restenosis rate of the target lesion) for DCB treatment of calcified lesions is also notably higher (90).

This may be attributed to calcified plaques impeding drug uptake and leading to insufficient local drug concentrations. Additionally, factors such as small vessel size (diameter <2.75 mm) and high thrombus burden (TIMI grade ≥3, with TIMI referring to the “Thrombolysis In Myocardial Infarction” blood flow grading standard) further reduce the efficacy of DCB in calcified ISR (91). Notably, DCB therapy guided by intravascular ultrasound (IVUS) can significantly reduce the risk of restenosis [Hazard Ratio (HR) 0.05], possibly related to optimized lesion preparation and ensuring adequate balloon apposition (90).

The main limitation of traditional treatment methods lies in their difficulty in effectively addressing the critical issue of stent under-expansion. Stent under-expansion is a significant predictor of ISR and is strongly associated with adverse events such as stent thrombosis and myocardial infarction (56). However, in cases of severe calcified lesions, even the use of high-pressure balloons (e.g., OPN balloons applying pressure up to 50 atm) may fail to achieve adequate expansion and may increase the risk of balloon rupture (92). Moreover, the micro-fragments generated during mechanical removal of calcifications may trigger distal embolization and the no-reflow phenomenon, further compromising treatment outcomes (93). Therefore, there is an urgent clinical need to develop safer and more effective techniques for addressing calcified lesions to improve the long-term prognosis for ISR patients.

### Extracorporeal shock wave lithotripsy (IVL)

Intravascular lithotripsy (IVL) is an emerging endovascular therapy that utilizes acoustic pressure waves to selectively disrupt calcified coronary plaques, thereby improving vessel compliance and facilitating optimal stent expansion (21). Its mechanism is based on the selective fracturing of calcific tissue, generating micro-cracks within the plaque while minimizing injury to the surrounding soft vascular wall (21, 94). This characteristic renders IVL an effective strategy for managing severely calcified lesions refractory to conventional balloon dilation or atherectomy. Clinically, IVL primarily targets two complex coronary scenarios: (1) stent underexpansion during initial implantation due to severe calcification, and (2) calcification-related in-stent restenosis (ISR) (72). In the former scenario, IVL directly modifies the calcific plaque to facilitate acute stent expansion. For ISR, it is particularly suitable for treating calcified neoatherosclerosis or external calcium compression of the stent, creating favorable conditions for subsequent therapy with drug-coated balloons or stent re-implantation (72).

IVL demonstrates significant efficacy and a favorable safety profile in coronary interventions. Clinical studies confirm its ability to substantially increase the minimum lumen area post-stent implantation, with one report reporting an increase from 3.88 mm^2^ to 7.41 mm^2^ on average (*p* < 0.001) (95). As a salvage therapy for underexpanded stents, IVL can increase the minimum lumen area by 238% (96). From a safety perspective, compared to rotational atherectomy, IVL is associated with shorter procedure times, reduced contrast volume, and lower complication rates (55). Real-world data indicate a high rate of procedural success (98% achieving TIMI grade 3 flow) with low rates of flow-limiting dissections (2.7%) and no reported perforations in one series (88). However, IVL's effectiveness varies depending on calcification morphology; optical coherence tomography (OCT) studies indicate that nodular (OR: 0.24) or thicker calcifications (OR: 0.66) are less responsive to treatment (72). Future directions involve optimizing treatment parameters guided by intravascular imaging and evaluating long-term outcomes through large registries (94).

### Emerging minimally invasive technologies

Emerging minimally invasive technologies offer novel mechanisms to address the limitations of conventional methods in treating coronary in-stent calcific restenosis. Laser balloon technology selectively ablates calcified tissue using precisely controlled laser energy, preserving the surrounding vascular wall; in contrast, ultrasound catheter technology fractures calcified plaques via high-frequency mechanical vibrations and offers real-time imaging guidance (21). Furthermore, cutting balloon technology, particularly advanced iterations such as RODIN-CUT, utilizes micro-blades integrated into the balloon surface to score and dilate calcified plaques in a controlled manner. The RODIN-CUT system incorporates a distinctive configuration of circumferentially arranged micro-blades that facilitate uniform and predictable plaque modification, allowing cutting depth to be modulated by balloon inflation pressure. This design enables a “single-step” procedure combining scoring and dilation, offering improved deliverability and controlled expansion in challenging in-stent restenosis (ISR) lesions, thereby achieving significant lumen gain (97, 98).Orbital atherectomy, a high-speed rotational technique, is particularly effective for nodular and severe concentric calcification, as it pulverizes plaque into microparticles with a lower perforation risk compared to traditional rotational atherectomy, evidenced by a 96.7% procedural success rate in calcified ISR (99, 100). Similarly, excimer laser coronary atherectomy (ELCA) employs ultraviolet light to ablate calcified and fibrotic plaques and has demonstrated high success rates in complex lesions, including those with thrombus burden (101). These technologies provide targeted, less traumatic alternatives for lesions resistant to standard balloon expansion.

The evolution of treatment strategies involves synergistic hybrid approaches and next-generation devices for personalized, precise intervention. Hybrid therapies combine modalities such as rotational or orbital atherectomy combined with intravascular lithotripsy (IVL), or excimer laser atherectomy paired with IVL to fragment complex calcification more effectively while minimizing vascular injury, often guided by intravascular imaging to match the specific calcification morphology (102). Concurrently, novel devices are enhancing deliverability and efficacy. Next-generation balloon-based IVL systems (e.g., Lithix) feature a lower profile and optimized energy delivery for tortuous vessels, demonstrating promising outcomes in stent expansion and low target lesion failure rates (103). The forward-directed mechanical modification system (e.g., Javelin) is designed for targeted ablation of protruding calcified nodules with real-time feedback, achieving high lesion preparation success rates (104). These advancements enable a tailored, minimally invasive strategy for high-risk and refractory coronary calcified lesions.

### New strategies for molecular targeted intervention

Emerging molecular targeted strategies for combating vascular calcification increasingly target the C5a-C5aR1-PERK-eIF2α-ATF4-CREB3L1 signaling axis as a pivotal therapeutic node, facilitating precise targeting of interconnected pathological drivers such as complement system activation and endoplasmic reticulum stress through agents like C5a/C5aR1 inhibitors and PERK pathway blockers (105–107). This approach is enhanced by the integration of synergistic interventions that operate via multiple mechanisms, including exerting potent anti-inflammatory effects, restoring compromised mitochondrial function, and restoring calcium-phosphorus homeostasis, while concurrently optimizing therapeutic potential through drug repurposing strategies for existing conventional medications (106, 107). Furthermore, comprehensive therapeutic frameworks are being developed that strategically integrate modulation of upstream signaling cascades with downstream effector pathways, as well as direct targeting of core pathological mechanisms with broader regulation of the tissue microenvironment, aiming to efficiently halt or reverse the progression of vascular calcification (105–107).

## Prognostic assessment and follow-up of in-stent calcific restenosis

### Importance of imaging follow-up

Imaging follow-up is crucial for the early identification, risk assessment, and decision-making regarding intervention timing for in-stent calcific restenosis. Regular use of intravascular imaging techniques such as Optical Coherence Tomography (OCT) and Intravascular Ultrasound (IVUS) can accurately assess the expansion status of the stent, wall apposition, and the dynamic evolution of calcific plaques, thereby providing key evidence for clinical management.

Dynamic monitoring of calcific plaque evolution aids in the timely detection of restenosis risk. Calcific nodules represent a unique calcific phenotype associated with a higher incidence of target lesion revascularization, characterized by an irregular eruptive surface, which particularly indicates poor prognosis (76). In addition, OCT follow-up can identify newly formed calcific nodules within the stent, a unique pattern of restenosis after stenting that may lead to a cycle of recurrent adverse interventions (76). Stent fracture is an important mechanical factor leading to late restenosis, especially in areas with severe calcification, vessel angulation, or persistent mechanical stress (34). Therefore, through a series of imaging examinations, such as OCT, ultrasound, and CT angiography, it is possible to track the progression of calcific plaques, the proliferation pattern of neointima, and the integrity of stent structures, achieving early warning of restenosis risk.

By integrating hemodynamic indicators for comprehensive assessment, physicians can make more evidence-based decisions regarding the timing of reintervention. The anatomical degree of stenosis alone is insufficient as an intervention criterion and must be combined with the patient's clinical symptoms and hemodynamic parameters. In managing stent under-expansion, IVUS and OCT can provide detailed assessments of the vascular and previously implanted stent lesions, clarifying the mechanisms of stent failure, thus guiding the selection of appropriate treatment strategies, including high-pressure balloon dilation, cutting balloon angioplasty, or calcific plaque atherectomy, alongside other calcification modification techniques (56). This individualized follow-up strategy, based on multimodal imaging and functional assessments, aims to balance the benefits and risks of intervention, avoiding unnecessary repeat procedures. Furthermore, this strategy ensures that when hemodynamically significant restenosis occurs, revascularization procedures can be performed promptly.

### Biomarkers and risk prediction

The mechanism of in-stent restenosis (ISR) is closely related to various biomarkers, among which inflammatory factors and calcium metabolism-related indicators hold significant value in risk assessment. Studies have shown that cysteine proteases (CTSs) play a critical role in the development of vascular diseases, with their expression and activity regulated by various stimuli such as inflammatory factors, oxidative stress, neurohormones, and growth factors (29). CTSs are not only implicated in pathophysiological processes including atherosclerosis, thrombosis, and calcification, but can also function as circulating and tissue-based biomarkers for assessing the risk of atherosclerotic cardiovascular disease (ACVD) (29). Furthermore, the C-reactive protein to albumin ratio (CAR) has also been confirmed to be significantly associated with ISR, with elevated CAR levels being an independent risk factor for restenosis after stenting (108). These biomarkers provide important evidence for early clinical identification of high-risk patients.

The future integration of multi-omics data is expected to facilitate precise early detection for ISR. Driven by advancements in omics technologies, the integrative analysis of multi-dimensional data such as genomics, proteomics, and metabolomics has become an area of intense research interest in recent years. For instance, long non-coding RNA (lncRNA) and miR-21 have shown good potential as molecular biomarkers in predicting coronary restenosis (109, 110). Additionally, machine learning-based predictive models (such as XGBoost) have incorporated 31 variables, including lymphocyte to monocyte ratio (LMR) and residual cholesterol (RC), yielding an AUC of 0.8098 for ISR prediction, thereby significantly outperforming traditional statistical methods (111). These innovative methods offer new research directions for early warning and precise intervention of ISR by deeply exploring the associations between biomarkers and clinical characteristics.

### Patient management and lifestyle intervention

The comprehensive management of patients with coronary artery disease is fundamental to preventing in-stent restenosis (ISR), extending beyond procedural success to address underlying risk factors. Strict control of metabolic diseases—including diabetes (target HbA1c < 7%), hypertension (target BP <130/80 mmHg), and dyslipidemia (target LDL-C < 1.4 mmol/L)—is paramount, as these conditions are strongly associated with the progression of coronary calcification and ISR risk; for instance, stringent glycemic control can reduce ISR incidence by 28% (112–114). This pharmacological strategy is synergistically supported by essential lifestyle interventions: complete smoking cessation, which can restore endothelial function and slow ISR progression; adherence to a Mediterranean diet rich in fiber and ω-3 fatty acids to reduce systemic inflammation; and regular moderate-intensity aerobic exercise (≥150 min/week) to improve endothelial repair and optimize hemodynamics at the stent site (115–117).

Medication regimens must be personalized to intensively manage residual risk and stabilize the coronary plaque environment. Lipid-lowering therapy forms the cornerstone of treatment, employing high-intensity statins, often combined with ezetimibe or PCSK9 inhibitors, to achieve very low LDL-C levels and exert pleiotropic anti-inflammatory effects, thereby reducing neointimal hyperplasia and calcified plaque volume (118). The antiplatelet strategy is tailored to balance ischemic and bleeding risks, with current evidence supporting a transition to P2Y12 inhibitor monotherapy (e.g., clopidogrel) after an initial period of dual antiplatelet therapy for high-bleeding-risk patients (119). Furthermore, novel agents such as SGLT2 inhibitors for diabetic patients and emerging therapies such as siRNA-based inclisiran for intensive lipid management offer additional mechanisms to mitigate cardiovascular risk and potentially interfere with the vascular calcification process, representing an advancement in personalized pharmacological prevention (118).

### Evidence-based standardized diagnostic and therapeutic algorithm for coronary in-stent calcific restenosis

**Figure 6:**
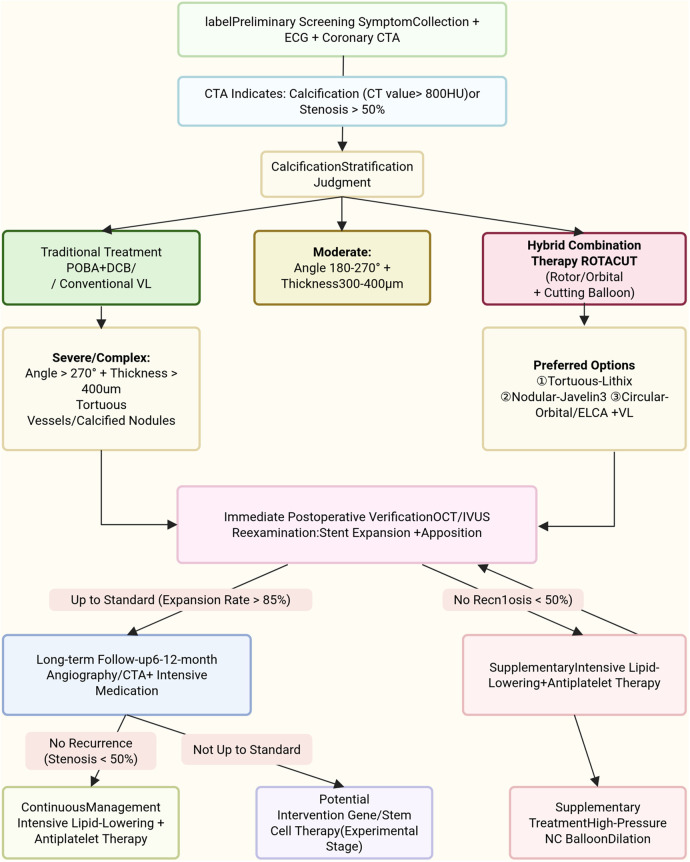
Evidence-based standardized diagnostic and therapeutic flowchart for coronary in-stent calcified restenosis (ISR). The flowchart consists of five key stages: ① Preliminary screening: Suspected cases are identified through symptom assessment, electrocardiograms, and coronary CTA (positive criteria: calcification with CT value >800 HU or luminal stenosis >50%); ② Precise assessment: OCT/IVUS is usemployed to decharacterminze calcification morphology (circumferential/eccentric/nodular), angle (<180°/180–270°/>270°), and thickness (<300 μm/300–400 μm/>400 μm); ③ Stratified treatment: Traditional regimens (POBA + DCB or conventional IVL) are selected for mild calcification, ROTACUT hybrid combination therapy is applied for moderate calcification, and novel devices such as Lithix/Javelin or Orbital/ELCA + IVL combination therapy are applied for severe/complex calcification (e.g., tortuous vessels, calcified nodules); ④ Immediate post-procedural verification: Stent expansion rate is re-evaluated by OCT/IVUS (≥85% as qualified), and cases failing to meet this criterion receive supplementary treatment with high-pressure NC balloon dilation; ⑤ Long-term follow-up: Coronary angiography/CTA is performed at 6–12 months; patients without recurrence continue intensive lipid-lowering therapy (LDL-C <1.4 mmol/L) and antiplatelet therapy, while gene/stem cell therapy (in the experimental phase) may be considered for recurrent/refractory cases.

(1). Preliminary Screening: The diagnostic pathway begins with clinical symptom assessment, electrocardiography (ECG), and coronary computed tomography angiography (CTA) to identify significant in-stent restenosis (stenosis >50%) and quantify the associated calcific burden (e.g., >800 Hounsfield Units), providing an initial anatomical roadmap (15). (2). Precise Assessment: This is followed by mandatory intravascular imaging—using optical coherence tomography (OCT) or intravascular ultrasound (IVUS)—for precise characterization of the calcification morphology (e.g., nodular vs. superficial), arc, thickness, and distribution, which is critical for therapeutic decision-making (75, 120, 121). (3). Stratified Treatment: Guided by imaging findings, a stratified treatment approach is implemented. For mild calcification, conventional plain old balloon angioplasty (POBA) combined with a drug-coated balloon (DCB) or standard intravascular lithotripsy (IVL) may suffice, given that adequate lesion preparation is key for DCB efficacy in calcified settings (122, 123). For moderate lesions, combined techniques like ROTACUT (rotational or orbital atherectomy followed by a cutting balloon) are employed to modify resistant calcium (124, 125). For severe or complex cases (e.g., tortuous anatomy, nodules, concentric calcium), advanced strategies such as next-generation low-profile IVL (e.g., Lithix), forward-directed atherectomy (e.g., Javelin for nodules), or hybrid approaches combining orbital/Excimer laser atherectomy with IVL are utilized based on their respective evidence bases for safety and efficacy in modifying challenging calcific plaques (104, 126–128). (4). Post-procedural Verification: Immediate procedural success is confirmed through repeat OCT/IVUS to ensure optimal stent expansion (e.g., expansion rate ≥85%), with adjunctive high-pressure balloon dilation performed if results are suboptimal, a step proven to mitigate the risk of restenosis associated with stent malapposition (129). (5). Long-term Follow-up: The algorithm culminates in structured surveillance, including repeat angiography or CTA at 6–12 months, coupled with intensive secondary prevention involving maximally tolerated lipid-lowering therapy [e.g., high-intensity statins, which reduce ISR risk (118)] and tailored antiplatelet regimens. Experimental options like gene or stem cell therapies are reserved for refractory cases (Figure 6).

## Future research directions and challenges

Future research directions for coronary in-stent calcified restenosis (ISR) will focus on four core areas: mechanistic research to clarify the molecular crosstalk between coronary calcification and neoatherosclerosis and to explore targeted anti-calcification agents such as BMP inhibitors and Lp(a) antagonists (130); imaging innovations leveraging AI-integrated OCT/IVUS to enable automatic calcification quantification and intraoperative real-time navigation (79, 131); treatment optimization through large-scale RCTs to validate the long-term efficacy of hybrid therapies (e.g., Orbital/ELCA + IVL) and novel devices (Lithix, Javelin) for coronary calcified ISR (21–23, 132); and the advancement of precision medicine to establish a risk prediction model integrating imaging findings, biomarkers (e.g., osteopontin, miR-203), and clinical characteristics for Asian populations (109, 110, 133), thereby facilitating more effective personalized prevention and treatment of complex calcified lesions.

## Conclusion

Coronary in-stent calcific restenosis (ISR) is a complex and dynamically evolving condition, driven by the interplay of molecular dysregulation, biomechanical stress from rigid plaques, and chronic inflammation (4, 8, 9, 14). This pathological complexity necessitates a paradigm shift from viewing calcification as a static endpoint to assessing its biological activity and mechanical stability over time. High-resolution intravascular imaging, particularly when utilizing optical coherence tomography (OCT) to detect micro-calcifications and intravascular ultrasound (IVUS) to assess vascular remodeling, is fundamental for precise lesion characterization and has been shown to improve interventional decision-making accuracy (72, 134). Imaging guidance enables the implementation of stratified, individualized treatment strategies that effectively address traditional limitations. These include hybrid approaches [e.g., orbital/Excimer laser atherectomy combined with intravascular lithotripsy (IVL), or ROTACUT] and novel devices (e.g., low-profile IVL like Lithix or forward-directed systems like Javelin), which synergistically modify calcific plaques to optimize stent expansion and drug delivery (19, 21–23). Notably, while IVL significantly improves stent expansion, its use, especially in severe circumferential calcification, necessitates the use of adjunctive strategies like distal embolization protection (23, 135). A comprehensive “full-cycle management” approach is essential, encompassing preoperative risk stratification using calcification scoring, intraoperative stepwise plaque modification and stenting, and postoperative intensive medical therapy including prolonged antiplatelet regimens and strict control of metabolic risk factors. Future research must prioritize elucidating the molecular crosstalk between calcification and neointimal hyperplasia, conducting long-term multicenter trials to validate the efficacy and safety of novel technologies, and developing population-specific (e.g., Asian) risk models that integrate imaging, biomarkers, and clinical data. Multidisciplinary collaboration is crucial to overcome current limitations, such as the lack of long-term data for some emerging technologies and standardized endpoint definitions across studies, thereby advancing from passively treating calcific complications to actively preventing their progression.

## Glossary

ISR: in-stent restenosis
OCT: optical coherence tomography
IVUS: intravascular ultrasound
IVL: intravascular lithotripsy
POBA: plain old balloon angioplasty
DCB: drug-coated balloon
VSMCs: vascular smooth muscle cells
BMP: bone morphogenetic protein
FGFR3: fibroblast growth factor receptor 3
NF-κB: nuclear factor-κB
CTSs: cysteine proteases
Lp(a): lipoprotein(a)
ECM: extracellular matrix
IL-1β: interleukin-1β
TNF-α: tumor necrosis factor-α
IL-6: interleukin-6
miR-221/222: MicroRNA-221/222
HU: hounsfield units
Runx2: runt-related transcription factor 2
MMPs: matrix metalloproteinases
FAI: fat attenuation index
DES: drug-eluting stent
DAMPs: damage-associated molecular patterns
PCI: percutaneous coronary intervention
RA: rotational atherectomy
HPBD: high-pressure balloon dilatation
CKD: chronic kidney disease
CAC: coronary artery calcification
CBCT: cone beam computed tomography
CTA: computed tomography angiography
VIE: virtual intravascular endoscopy
PCD-CT: photon-counting detector CT
UHR-Bv72: ultra-high resolution Bv72 imaging
AUC: area under the curve
CAR: C-reactive protein/albumin ratio
MACE: major adverse cardiovascular events
TLR: target lesion revascularization
TIMI: thrombolysis in myocardial infarction
MSA: minimum lumen area
VEGF: vascular endothelial growth factor
TGF-β: transforming growth factor-β
LDL-C: low-density lipoprotein cholesterol
MGP: matrix gla protein
Osterix Osterix: (osteoblast-specific transcription factor)
ICAC: intracranial artery calcification
NLR: neutrophil-lymphocyte ratio
PLR: platelet-lymphocyte ratio
SII: systemic immune-inflammation index
lncRNA: long non-coding RNA
LMR: lymphocyte-monocyte ratio
RC: residual cholesterol
HbA1c: glycated hemoglobin
DAPT: dual antiplatelet therapy; PCSK9, proprotein convertase subtilisin/kexin type 9
ISC: in-stent calcification
ALP: alkaline phosphatase
EndMT: endothelial-mesenchymal transition
FEM: finite element model
PACSS: peripheral arterial calcification scoring system
DCB-PTA: paclitaxel drug-coated balloon percutaneous transluminal angioplasty
CDTLR: clinically driven target lesion revascularization
SECS: self-expanding covered stent
IWS: interwoven nitinol stent
ELCA: excimer laser coronary atherectomy
